# The influence of education on health: an empirical assessment of OECD countries for the period 1995–2015

**DOI:** 10.1186/s13690-020-00402-5

**Published:** 2020-04-06

**Authors:** Viju Raghupathi, Wullianallur Raghupathi

**Affiliations:** 1 Koppelman School of Business, Brooklyn College of the City University of New York, 2900 Bedford Ave, Brooklyn, NY 11210 USA; 2 Gabelli School of Business, Fordham University, 140 W. 62nd Street, New York, NY 10023 USA

**Keywords:** Health, Education level, Enrollment rate, Analytics, Life expectancy, Potential years of life lost, NEET, OECD, Infant mortality, Deaths from cancer

## Abstract

**Background:**

A clear understanding of the macro-level contexts in which education impacts health is integral to improving national health administration and policy. In this research, we use a visual analytic approach to explore the association between education and health over a 20-year period for countries around the world.

**Method:**

Using empirical data from the OECD and the World Bank for 26 OECD countries for the years 1995–2015, we identify patterns/associations between education and health indicators. By incorporating pre- and post-educational attainment indicators, we highlight the dual role of education as both a driver of opportunity as well as of inequality.

**Results:**

Adults with higher educational attainment have better health and lifespans compared to their less-educated peers. We highlight that tertiary education, particularly, is critical in influencing infant mortality, life expectancy, child vaccination, and enrollment rates. In addition, an economy needs to consider potential years of life lost (premature mortality) as a measure of health quality.

**Conclusions:**

We bring to light the health disparities across countries and suggest implications for governments to target educational interventions that can reduce inequalities and improve health. Our country-level findings on NEET (Not in Employment, Education or Training) rates offer implications for economies to address a broad array of vulnerabilities ranging from unemployment, school life expectancy, and labor market discouragement. The health effects of education are at the grass roots-creating better overall self-awareness on personal health and making healthcare more accessible.

## Introduction

Is education generally associated with good health? There is a growing body of research that has been exploring the influence of education on health. Even in highly developed countries like the United States, it has been observed that adults with lower educational attainment suffer from poor health when compared to other populations [36]. This pattern is attributed to the large health inequalities brought about by education. A clear understanding of the health benefits of education can therefore serve as the key to reducing health disparities and improving the well-being of future populations. Despite the growing attention, research in the education–health area does not offer definitive answers to some critical questions. Part of the reason is the fact that the two phenomena are interlinked through life spans within and across generations of populations [36], thereby involving a larger social context within which the association is embedded. To some extent, research has also not considered the variances in the education–health relationship through the course of life or across birth cohorts [20], or if there is causality in the same. There is therefore a growing need for new directions in education–health research.

The avenues through which education affects health are complex and interwoven. For one, at the very outset, the distribution and content of education changes over time [20]. Second, the relationship between the mediators and health may change over time, as healthcare becomes more expensive and/or industries become either more, or less hazardous. Third, some research has documented that even relative changes in socioeconomic status (SES) can affect health, and thus changes in the distribution of education implies potential changes in the relationship between education and health. The relative index of inequality summarizes the magnitude of SES as a source of inequalities in health [11, 21, 27, 29]. Fourth, changes in the distribution of health and mortality imply that the paths to poor health may have changed, thereby affecting the association with education.

Research has proposed that the relationship between education and health is attributable to three general classes of mediators: economic; social, psychological, and interpersonal; and behavioral health [31]. Economic variables such as income and occupation mediate the relationship between education and health by controlling and determining access to acute and preventive medical care [1, 2, 19]. Social, psychological, and interpersonal resources allow people with different levels of education to access coping resources and strategies [10, 34], social support [5, 22], and problem-solving and cognitive abilities to handle ill-health consequences such as stress [16]. Healthy behaviors enable educated individuals to recognize symptoms of ill health in a timely manner and seek appropriate medical help [14, 35].

While the positive association between education and health has been established, the explanations for this association are not [31]. People who are well educated experience better health as reflected in the high levels of self-reported health and low levels of morbidity, mortality, and disability. By extension, low educational attainment is associated with self-reported poor health, shorter life expectancy, and shorter survival when sick. Prior research has suggested that the association between education and health is a complicated one, with a range of potential indicators that include (but are not limited to) interrelationships between demographic and family background indicators [8] - effects of poor health in childhood, greater resources associated with higher levels of education, appreciation of good health behaviors, and access to social networks. Some evidence suggests that education is strongly linked to health determinants such as preventative care [9]. Education helps promote and sustain healthy lifestyles and positive choices, nurture relationships, and enhance personal, family, and community well-being. However, there are some adverse effects of education too [9]. Education may result in increased attention to preventive care, which, though beneficial in the long term, raises healthcare costs in the short term. Some studies have found a positive association between education and some forms of illicit drug and alcohol use. Finally, although education is said to be effective for depression, it has been found to have much less substantial impact in general happiness or well-being [9].

On a universal scale, it has been accepted that several social factors outside the realm of healthcare influence the health outcomes [37]. The differences in morbidity, mortality and risk factors in research, conducted within and between countries, are impacted by the characteristics of the physical and social environment, and the structural policies that shape them [37]. Among the developed countries, the United States reflects huge disparities in educational status over the last few decades [15, 24]. Life expectancy, while increasing for all others, has decreased among white Americans without a high school diploma - particularly women [25, 26, 32]. The sources of inequality in educational opportunities for American youth include the neighborhood they live in, the color of their skin, the schools they attend, and the financial resources of their families. In addition, the adverse trends in mortality and morbidity brought on by opioids resulting in suicides and overdoses (referred to as deaths of despair) exacerbated the disparities [21]. Collectively, these trends have brought about large economic and social inequalities in society such that the people with more education are likely to have more health literacy, live longer, experience better health outcomes, practice health promoting behaviors, and obtain timely health checkups [21, 17].

Education enables people to develop a broad range of skills and traits (including cognitive and problem-solving abilities, learned effectiveness, and personal control) that predispose them towards improved health outcomes [23], ultimately contributing to human capital. Over the years, education has paved the way for a country’s financial security, stable employment, and social success [3]. Countries that adopt policies for the improvement of education also reap the benefits of healthy behavior such as reducing the population rates of smoking and obesity. Reducing health disparities and improving citizen health can be accomplished only through a thorough understanding of the health benefits conferred by education.

There is an iterative relationship between education and health. While poor education is associated with poor health due to income, resources, healthy behaviors, healthy neighborhood, and other socioeconomic factors, poor health, in turn, is associated with educational setbacks and interference with schooling through difficulties with learning disabilities, absenteeism, or cognitive disorders [30]. Education is therefore considered an important social determinant of health. The influence of national education on health works through a variety of mechanisms. Generally, education shows a relationship with self-rated health, and thus those with the highest education may have the best health [30]. Also, health-risk behaviors seem to be reduced by higher expenditure into the publicly funded education system [18], and those with good education are likely to have better knowledge of diseases [33]. In general, the education–health gradients for individuals have been growing over time [38].

To inform future education and health policies effectively, one needs to observe and analyze the opportunities that education generates during the early life span of individuals. This necessitates the adoption of some fundamental premises in research. Research must go beyond pure educational attainment and consider the associated effects preceding and succeeding such attainment. Research should consider the variations brought about by the education–health association across place and time, including the drivers that influence such variations [36].

In the current research, we analyze the association between education and health indicators for various countries using empirical data from reliable sources such as the Organization for Economic Cooperation and Development (OECD) and World Bank. While many studies explore the relationship between education and health at a conceptual level, we deploy an empirical approach in investigating the patterns and relationships between the two sets of indicators. In addition, for the educational indicators, we not only incorporate the level of educational attainment, but also look at the potential socioeconomic benefits, such as enrollment rates (in each sector of educational level) and school life expectancy (at each educational level). We investigate the influences of educational indicators on national health indicators of infant mortality, child vaccinations, life expectancy at birth, premature mortality arising from lack of educational attainment, employment and training, and the level of national health expenditure. Our research question is:

*What are some key influencers/drivers in the education-health relationship at a country level?*

The current study is important because policy makers have an increasing concern on national health issues and on policies that support it. The effect of education is at the root level—creating better overall self-awareness on personal health and making healthcare more accessible. The paper is organized as follows: Section 2 discusses the background for the research. Section 3 discusses the research method; Section 4 offers the analysis and results; Section 5 provides a synthesis of the results and offers an integrated discussion; Section 6 contains the scope and limitations of the research; Section 7 offers conclusions with implications and directions for future research.

## Background

Research has traditionally drawn from three broad theoretical perspectives in conceptualizing the relationship between education and health. The majority of research over the past two decades has been grounded in the *Fundamental Cause Theory* (FCT) [28], which posits that factors such as education are fundamental social causes of health inequalities because they determine access to resources (such as income, safe neighborhoods, or healthier lifestyles) that can assist in protecting or enhancing health [36]. Some of the key social resources that contribute to socioeconomic status include education (knowledge), money, power, prestige, and social connections. As some of these undergo change, they will be associated with differentials in the health status of the population [12].

Education has also been conceptualized using the *Human Capital Theory* (HCT) that views it as a return on investment in the form of increased productivity [4]. Education improves knowledge, skills, reasoning, effectiveness, and a broad range of other abilities that can be applied to improving health. The third approach - the *signaling or credentialing perspective* [6] - is adopted to address the large discontinuities in health at 12 and 16 years of schooling, which are typically associated with the receipt of a high school diploma and a college degree, respectively. This perspective considers the earned credentials of a person as a potential source that warrants social and economic returns. All these theoretical perspectives postulate a strong association between education and health and identify mechanisms through which education influences health. While the HCT proposes the mechanisms as embodied skills and abilities, FCT emphasizes the dynamism and flexibility of mechanisms, and the credentialing perspective proposes educational attainment through social responses. It needs to be stated, however, that all these approaches focus on education solely in terms of attainment, without emphasizing other institutional factors such as quality or type of education that may independently influence health. Additionally, while these approaches highlight the individual factors (individual attainment, attainment effects, and mechanisms), they do not give much emphasis to the social context in which education and health processes are embedded.

In the current research while we acknowledge the tenets of these theoretical perspectives, we incorporate the social mechanisms in education such as level of education, skills and abilities brought about by enrollment, school life expectancy, and the potential loss brought about by premature mortality. In this manner, we highlight the relevance of the social context in which the education and health domains are situated. We also study the dynamism of the mechanisms over countries and over time and incorporate the influences that precede and succeed educational attainment.

## Methods

We analyze country level education and health data from the OECD and World Bank for a period of 21 years (1995–2015). Our variables include the education indicators of adult education level; enrollment rates at various educational levels; NEET (Not in Employment, Education or Training) rates; school life expectancy; and the health indicators of infant mortality, child vaccination rates, deaths from cancer, life expectancy at birth, potential years of life lost and smoking rates (Table 1). The data was processed using the tools of Tableau for visualization, and SAS for correlation and descriptive statistics. Approaches for analysis include ranking, association, and data visualization of the health and education data.

**Table 1:** Variables in the Research

Category	Variable	Description
Education	Primary School Enrollment Rate-Gross (ratio)	The ratio of total enrollment (regardless of age) to the population of the age group in the level of education shown. It is estimated as a weighted average. Note: Gross enrollment includes students of all ages for a level. Therefore, a high ratio may reflect a large number of overage children enrolled in each level due to repetition or late entry. This number can exceed the official population of students in the age group for the education level. This can lead to ratios above 100%.
Education	Secondary School Enrollment Rate-Gross (ratio)	
Education	Tertiary School Enrollment Rate-Gross (ratio)	
Education	Tertiary School Life Expectancy (Years)	The total number of years of tertiary schooling that a child can expect to receive.
Education	Adult Education Level Below Secondary (ratio)	The highest level of education completed by the 25–64-year-old population.
Education	Adult Education Level Upper Secondary (ratio)	
Education	Adult Education Level Tertiary (ratio)	
Education	NEET Rate [Age: 15–29] (%)	Those who are not in employment, education, or training (NEET), as a % of the total number in the corresponding age group.
Education	NEET Rate [Age: 15–19] (%)	
Education	NEET Rate [Age: 20–24] (%)	
Control	GDP per capita ($)	Gross domestic product per person.
Control	GDP per capita (group) (categories)	Classification of countries by GDP into high, medium and low
Control	GDP per capita (bin) ($)	Countries classified within each GDP group into categories in intervals of 10 K ranging from 0 K–110 K
Control	Continents and Countries	Continents: Asia (AS), Europe (EU), Oceania (OA), North America (NA) and South America (SA) Countries included in the above continents: Israel, Korea, Belgium, Switzerland, Czeck Republic, Denmark, Spain, Finland, France, Great Britain, Greece, Hungary, Ireland, Iceland, Italy, Luxembourg, Netherlands, Norway, Poland, Portugal, Sweden, Turkey, United States of America, Australia, Costa Rica, Mexico
Health	Compulsory Health Expenditure ($)	Current health expenditure including personal health care &collective services but excluding spending on investments.
Health	Life Expectancy at Birth (Years)	How long, on average, a newborn can expect to live, if current death rates do not change
Health	Potential Year of Life Lost (Years lost per 100,000 persons, aged 0–69)	Summary measure of premature mortality, providing an explicit way of weighting deaths occurring at younger ages, which may be preventable.
Health	Deaths from Cancer (Deaths per 100,000 persons)	Numbers of deaths registered in a country in a year divided by the size of the corresponding population.
Health	Infant Mortality Rate (per 1000 live births)	The number of deaths of children under one year of age, expressed per 1000 live births
Health	Smoking Rate (%)	% of the population aged 15 years and over who report smoking every day.
Health	Rate of Child Vaccination [Age: 15–19] (%)	Percentage of children who receive the respective vaccination in the recommended timeframe.
Health	Rate of Child Vaccination [20–24] (%)	

## Analyses and results

In this section we identify and analyze patterns and associations between education and health indicators and discuss the results. Since countries vary in population sizes and other criteria, we use the estimated averages in all our analyses.

### Comparison of health outcomes for countries by GDP per capita

**Fig. 1:**
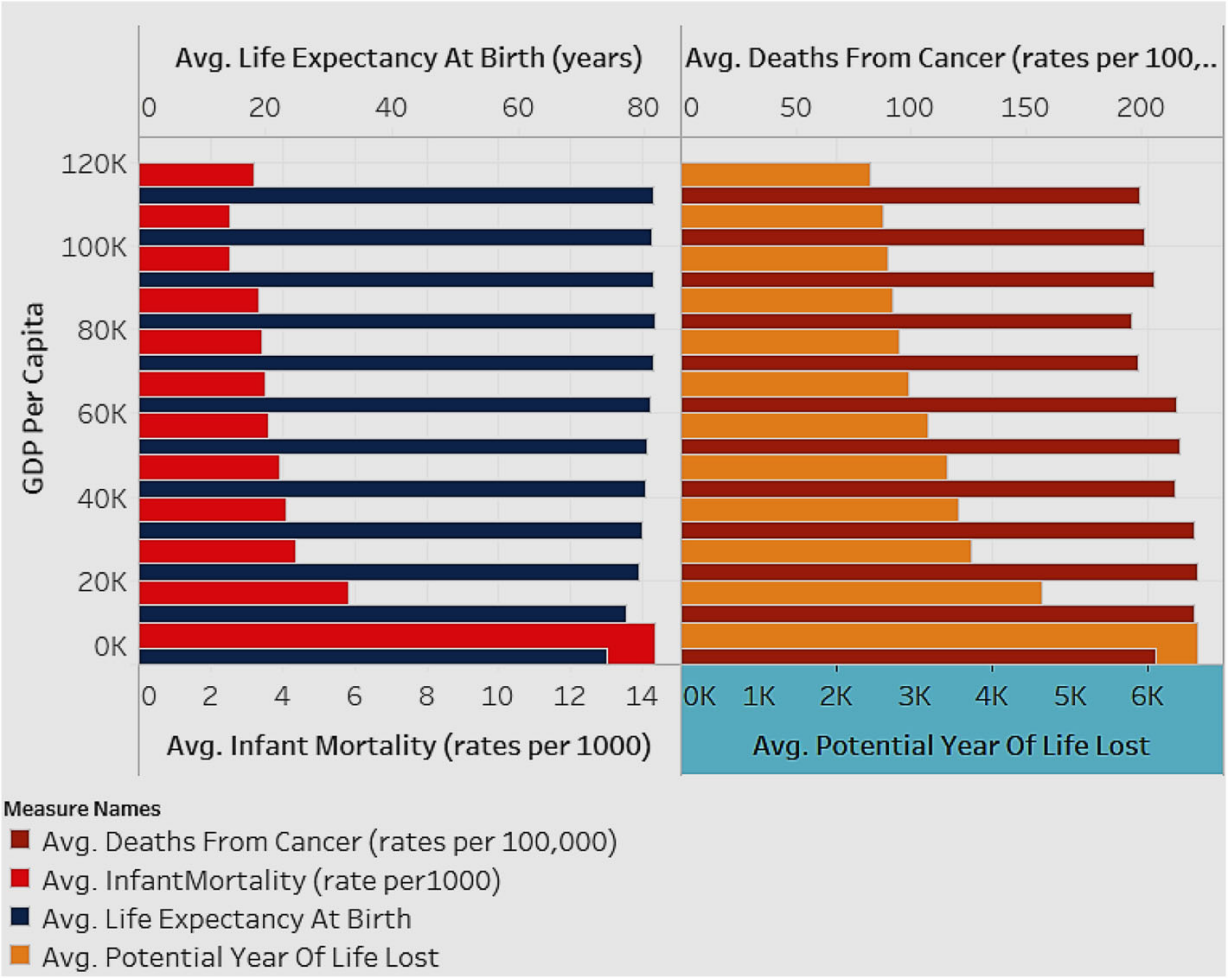
Associations between Average Life Expectancy (years) and Average Infant Mortality rate (per 1000), and between Deaths from Cancer (rates per 100,000) and Average Potential Years of Life Lost (years), by GDP per capita (for all countries for years 1995–2015)

We first analyzed to see if our data reflected the expectation that countries with higher GDP per capita have better health status (Fig. 1). We compared the average life expectancy at birth, average infant mortality, average deaths from cancer and average potential year of life lost, for different levels of GDP per capita (Fig. 1).

Figure 1 depicts two charts with the estimated averages of variables for all countries in the sample. The X-axis of the first chart depicts average infant mortality rate (per 1000), while that of the second chart depicts average potential years of life lost (years). The Y-axis for both charts depicts the GDP per capita shown in intervals of 10 K ranging from 0 K–110 K (US Dollars). The analysis is shown as an average for all the countries in the sample and for all the years (1995–2015). As seen in Fig. 1, countries with lower GDP per capita have higher infant mortality rate and increased potential year of life lost (which represents the average years a person would have lived if he or she had not died prematurely - a measure of premature mortality). Life expectancy and deaths from cancer are not affected by GDP level. When studying infant mortality and potential year lost, in order to avoid the influence of a control variable, it was necessary to group the samples by their GDP per capita level.

### Association of Infant Mortality Rates with enrollment rates and education levels

**Fig. 2:**
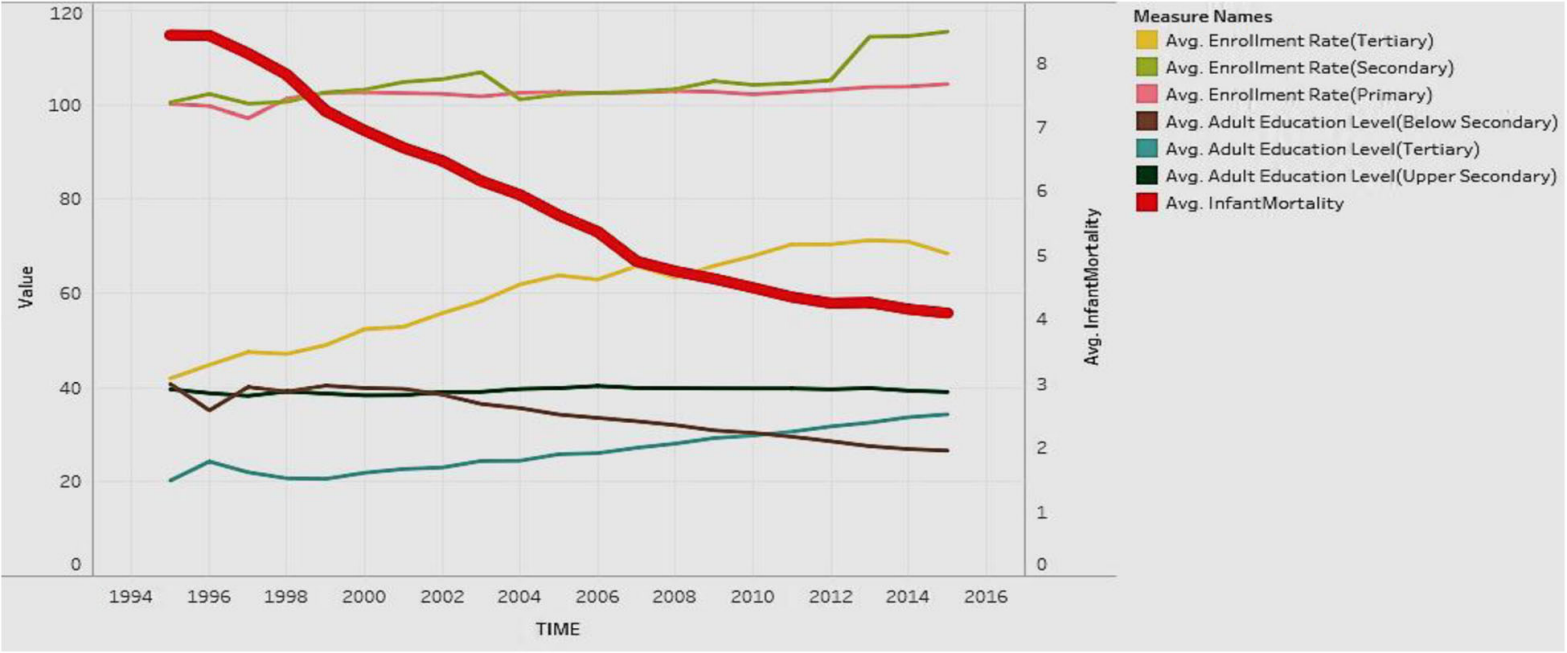
Association of Adult Education Levels (ratio) and Enrollment Rates (ratio) with Infant Mortality Rate (per 1000)

We explored the association of infant mortality rates with the enrollment rates and adult educational levels for all countries (Fig. 2). The expectation is that with higher education and employment the infant mortality rate decreases.

Figure 2 depicts the analysis for all countries in the sample. The figure shows the years from 1995 to 2015 on the X axis. It shows two Y-axes with one axis denoting average infant mortality rate (per 1000 live births), and the other showing the rates from 0 to 120 to depict enrollment rates (primary/secondary/tertiary) and education levels (below secondary/upper secondary/tertiary). Regarding the Y axis showing rates over 100, it is worth noting that the enrollment rates denote a ratio of the total enrollment (regardless of age) at a level of education to the official population of the age group in that education level. Therefore, it is possible for the number of children enrolled at a level to exceed the official population of students in the age group for that level (due to repetition or late entry). This can lead to ratios over 100%. The figure shows that in general, all education indicators tend to rise over time, except for adult education level below secondary, which decreases over time. Infant mortality shows a steep decreasing trend over time, which is favorable. In general, countries have increasing health status and education over time, along with decreasing infant mortality rates. This suggests a negative association of education and enrollment rates with mortality rates.

### Association of Education Outcomes with life expectancy at birth

**Fig. 3:**
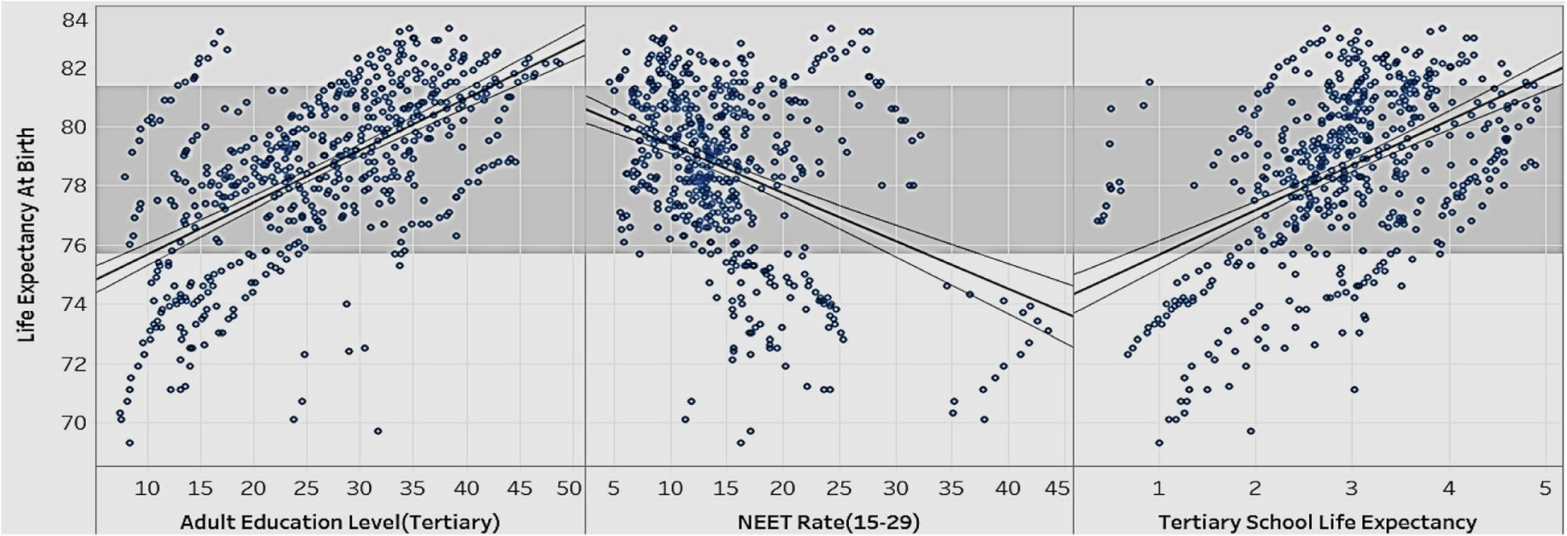
Association of Adult Education Level (Tertiary), NEET rate, School Life Expectancy (Tertiary), with Life Expectancy at Birth

We explored if the education outcomes of adult education level (tertiary), school life expectancy (tertiary), and NEET (not in employment, education, or training) rates, affected life expectancy at birth (Fig. 3). Our expectation is that adult education and school life expectancy, particularly tertiary, have a positive influence, while NEET has an adverse influence, on life expectancy at birth.

Figure 3 show the relationships between various education indicators (adult education level-tertiary, NEET rate, school life expectancy-tertiary) and life expectancy at birth for all countries in the sample. The figure suggests that life expectancy at birth rises as adult education level (tertiary) and tertiary school life expectancy go up. Life expectancy at birth drops as the NEET rate goes up. In order to extend people’s life expectancy, governments should try to improve tertiary education, and control the number of youths dropping out of school and ending up unemployed (the NEET rate).

### Association of Tertiary Enrollment and Education with potential years of life lost

**Fig. 4:**
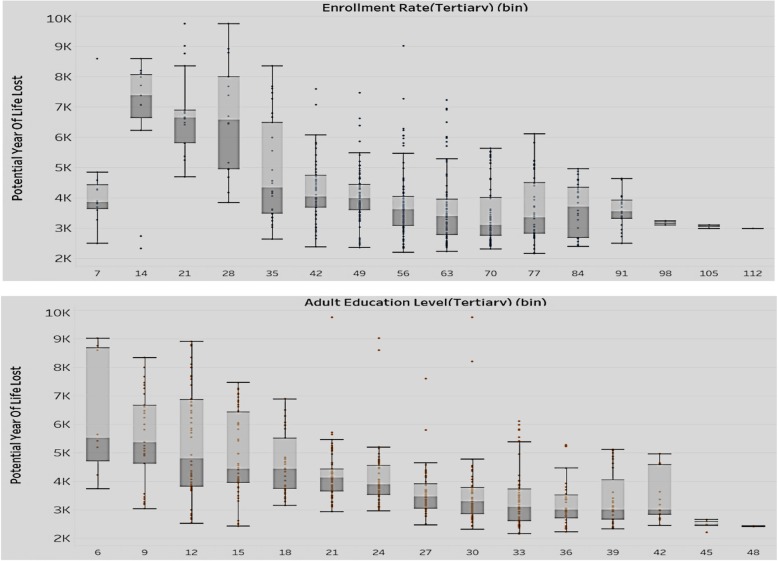
Association of Enrollment rate-tertiary (top) and Adult Education Level-Tertiary (bottom) with Potential Years of Life Lost (Y axis)

We wanted to explore if the potential years of life lost rates are affected by tertiary enrollment rates and tertiary adult education levels (Fig. 4).

The two sets of box plots in Fig. 4 compare the enrollment rates with potential years of life lost (above set) and the education level with potential years of life lost (below set). The analysis is for all countries in the sample. As mentioned earlier, the enrollment rates are expressed as ratios and can exceed 100% if the number of children enrolled at a level (regardless of age) exceed the official population of students in the age group for that level. Potential years of life lost represents the average years a person would have lived, had he/she not died prematurely. The results show that with the rise of tertiary adult education level and tertiary enrollment rate, there is a decrease in both value and variation of the potential years of life lost. We can conclude that lower levels in tertiary education adversely affect a country’s health situation in terms of premature mortality.

### Association of Tertiary Enrollment and Education with child vaccination rates

**Fig. 5:**
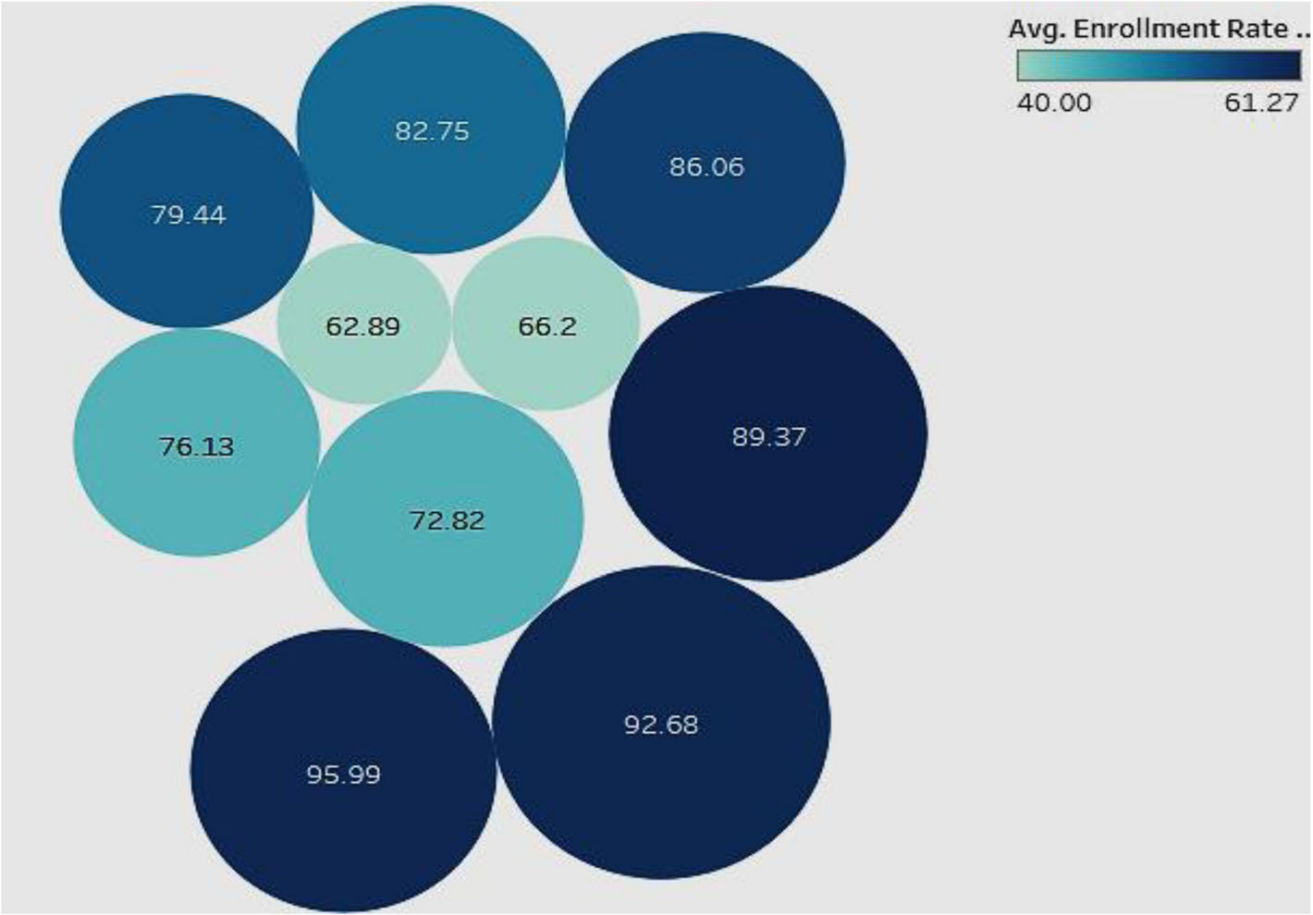
Association of Adult Education Level-Tertiary and Enrollment Rate-Tertiary with Child Vaccination Rates

We compared the performance of tertiary education level and enrollment rates with the child vaccination rates (Fig. 5) to assess if there was a positive impact of education on preventive healthcare.

In this analysis (Fig. 5), we looked for associations of child vaccination rates with tertiary enrollment and tertiary education. The analysis is for all countries in the sample. The color of the bubble represents the tertiary enrollment rate such that the darker the color, the higher the enrollment rate, and the size of the bubble represents the level of tertiary education. The labels inside the bubbles denote the child vaccination rates. The figure shows a general positive association of high child vaccination rate with tertiary enrollment and tertiary education levels. This indicates that countries that have high child vaccination rates tend to be better at tertiary enrollment and have more adults educated in tertiary institutions. Therefore, countries that focus more on tertiary education and enrollment may confer more health awareness in the population, which can be reflected in improved child vaccination rates.

### Association of NEET rates (15–19; 20–24) with infant mortality rates and deaths from Cancer

**Fig. 6:**
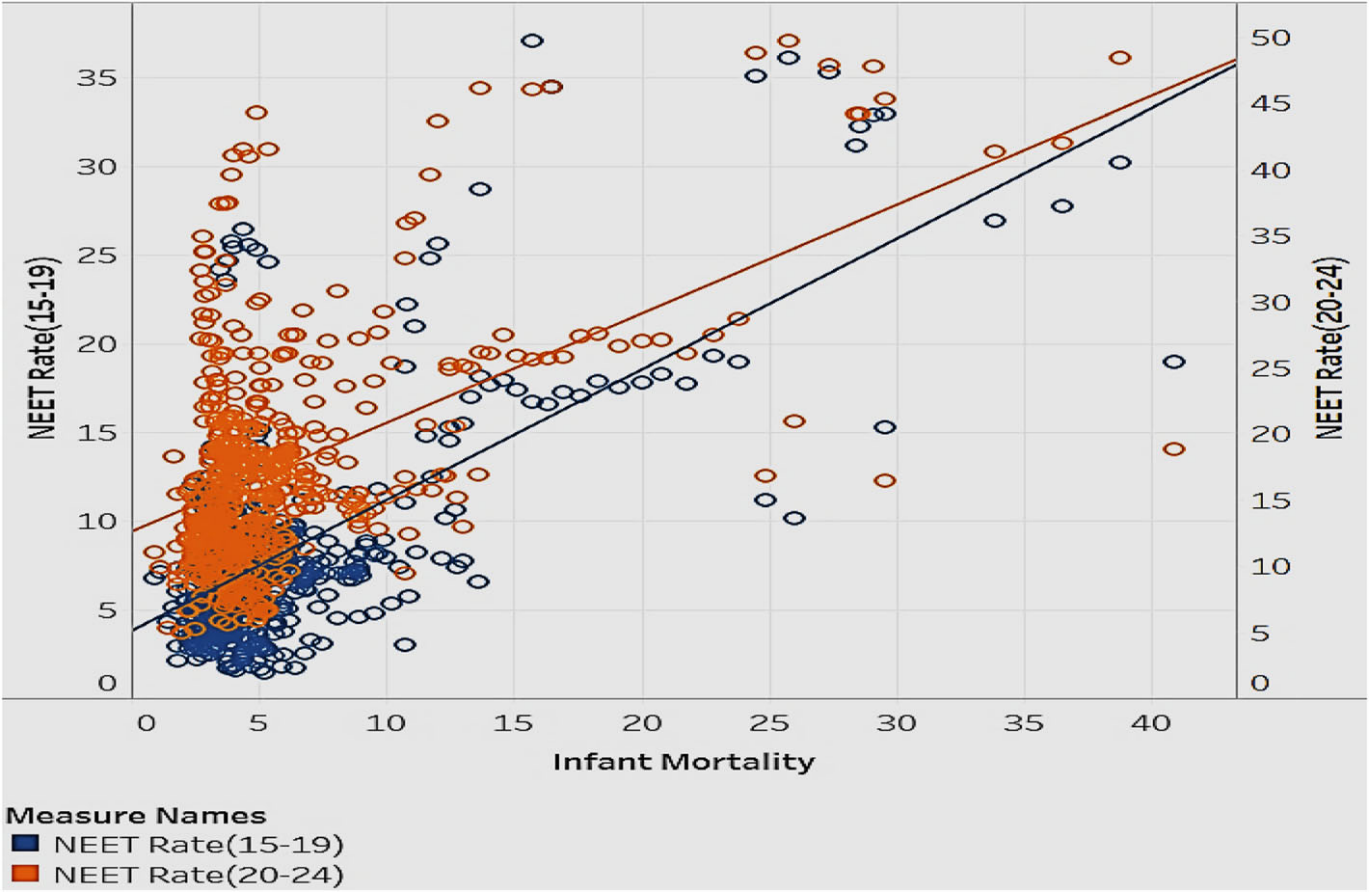
Association of Infant Mortality rates with NEET Rates (15–19) and NEET Rates (20–24)

In the realm of child health, we also looked at the infant mortality rates. We explored if infant mortality rates are associated with the NEET rates in different age groups (Fig. 6).

Figure 6 is a scatterplot that explores the correlation between infant mortality and NEET rates in the age groups 15–19 and 20–24. The data is for all countries in the sample. Most data points are clustered in the lower infant mortality and lower NEET rate range. Infant mortality and NEET rates move in the same direction—as infant mortality increases/decrease, the NEET rate goes up/down. The NEET rate for the age group 20–24 has a slightly higher infant mortality rate than the NEET rate for the age group 15–19. This implies that when people in the age group 20–24 are uneducated or unemployed, the implications on infant mortality are higher than in other age groups. This is a reasonable association, since there is the potential to have more people with children in this age group than in the teenage group. To reduce the risk of infant mortality, governments should decrease NEET rates through promotional programs that disseminate the benefits of being educated, employed, and trained [7]. Additionally, they can offer financial aid to public schools and companies to offer more resources to raise general health awareness in people.

**Fig. 7:**
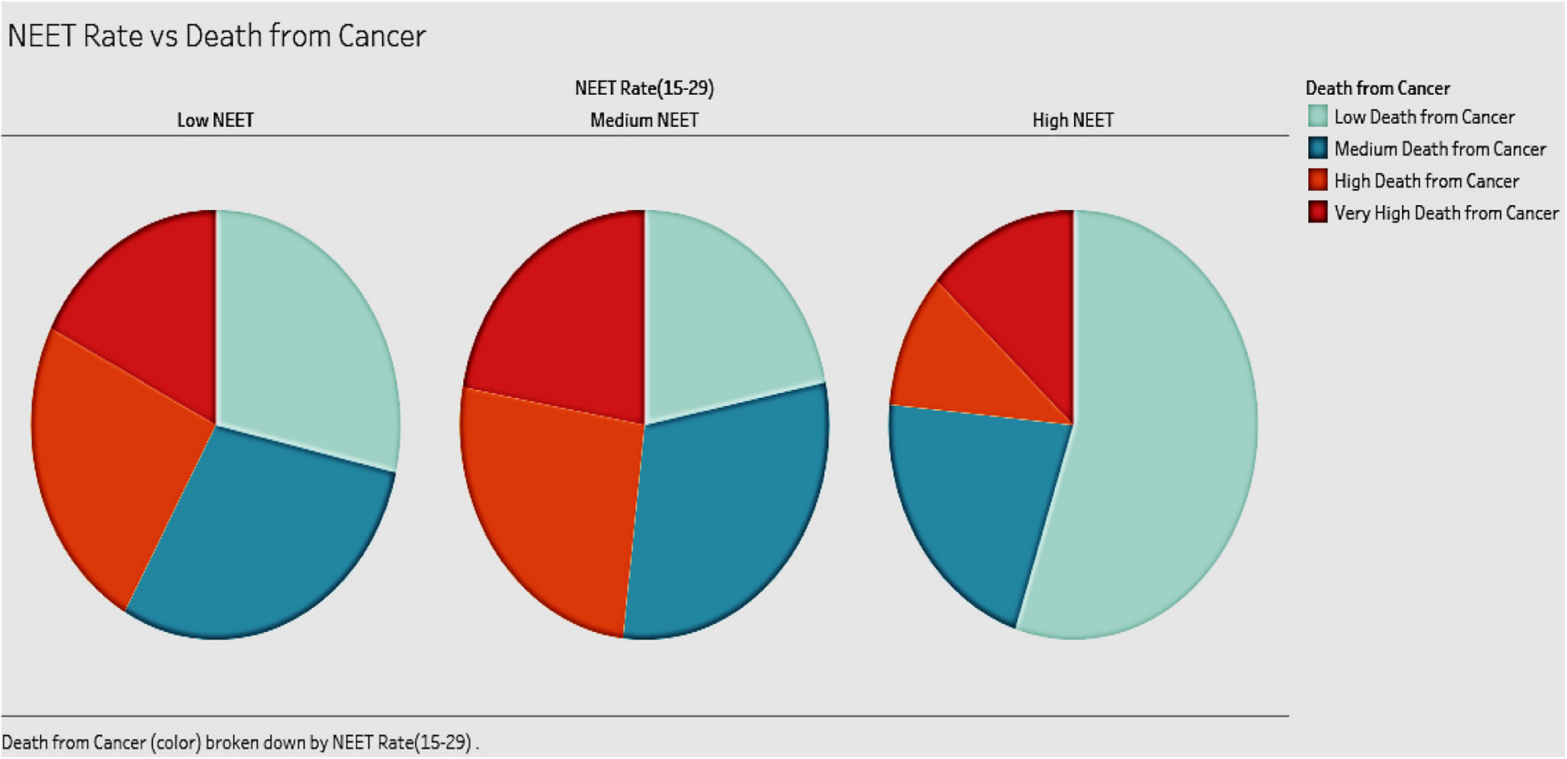
Association of Deaths from Cancer and different NEET Rates

We looked to see if the distribution of population without employment, education, or training (NEET) in various categories of high, medium, and low impacted the rate of deaths from cancer (Fig. 7). Our expectation is that high rates of NEET will positively influence deaths from cancer.

The three pie charts in Fig. 7 show the distribution of deaths from cancer in groups of countries with different NEET rates (high, medium, and low). The analysis includes all countries in the sample. The expectation was that high rates of NEET would be associated with high rates of cancer deaths. Our results, however, show that countries with medium NEET rates tend to have the highest deaths from cancer. Countries with high NEET rates have the lowest deaths from cancer among the three groups. Contrary to expectations, countries with low NEET rates do not show the lowest death rates from cancer. A possible explanation for this can be attributed to the fact that in this group, the people in the labor force may be suffering from work-related hazards including stress, that endanger their health.

### Association between adult education levels and health expenditure

**Fig. 8:**
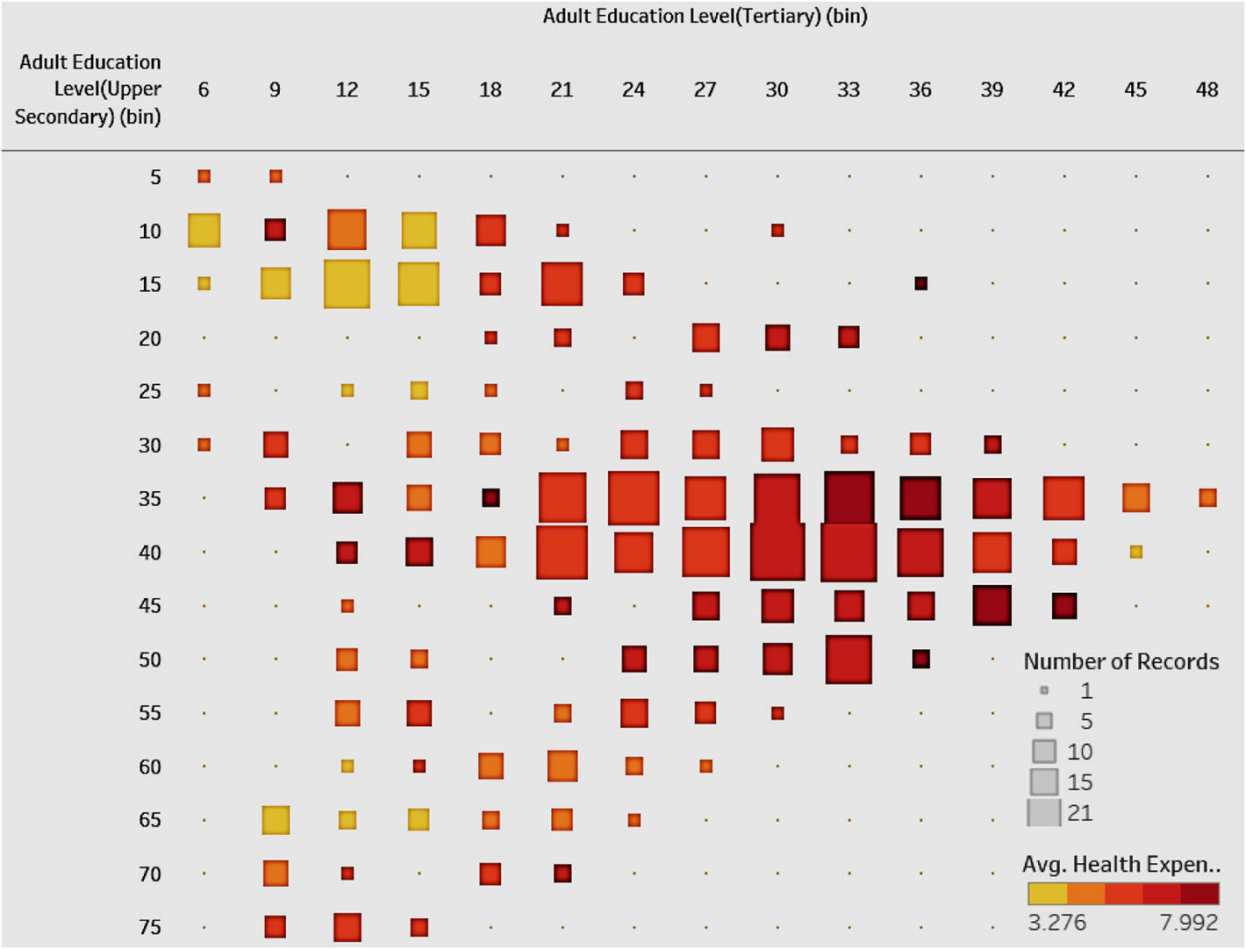
Association of Health Expenditure and Adult Education Level-Tertiary & Upper Secondary

It is interesting to note the relationship between health expenditure and adult education levels (Fig. 8). We expect them to be positively associated.

Figure 8 shows a heat map with the number of countries in different combinations of groups between tertiary and upper-secondary adult education level. We emphasize the higher levels of adult education. The color of the square shows the average of health expenditure. The plot shows that most of the countries are divided into two clusters. One cluster has a high tertiary education level as well as a high upper-secondary education level and it has high average health expenditure. The other cluster has relatively low tertiary and upper secondary education level with low average health expenditure. Overall, the figure shows a positive correlation between adult education level and compulsory health expenditure. Governments of countries with low levels of education should allocate more health expenditure, which will have an influence on the educational levels. Alternatively, to improve public health, governments can frame educational policies to improve the overall national education level, which then produces more health awareness, contributing to national healthcare.

### Association of Compulsory Health Expenditure with NEET rates by country and region

**Fig. 9:**
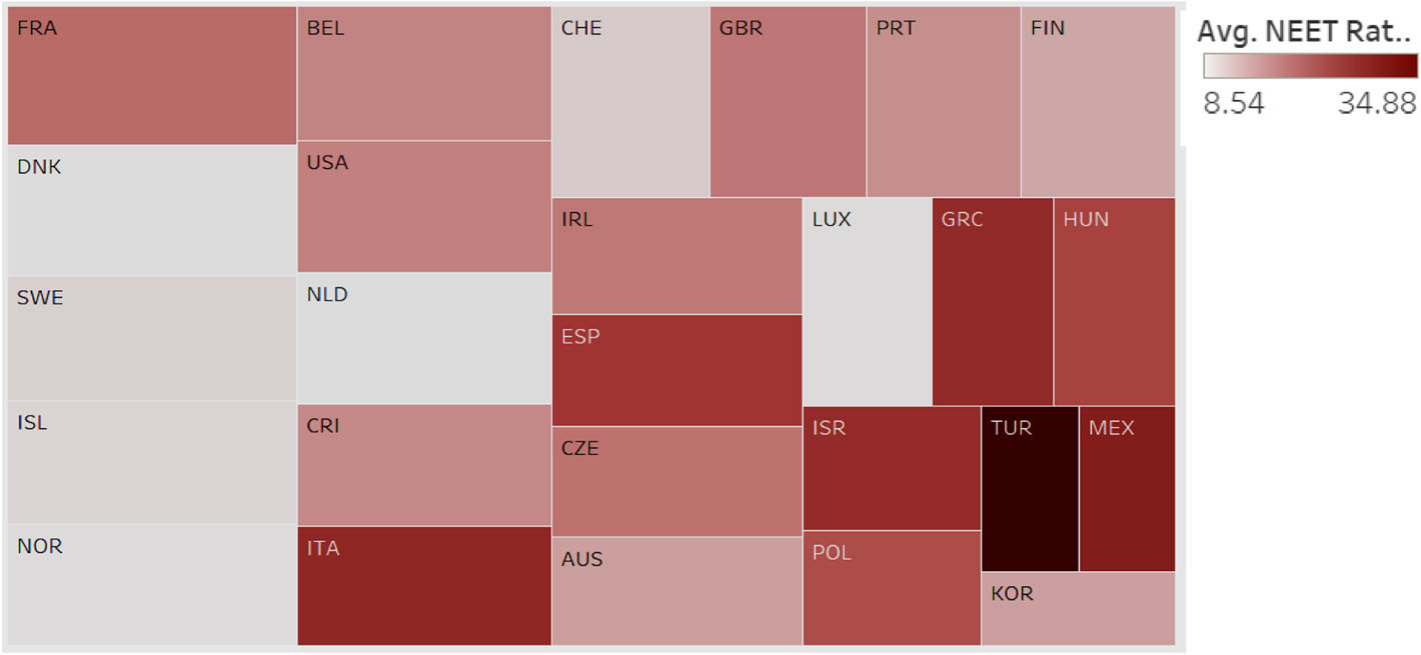
Association between Compulsory Health Expenditure and NEET Rate by Country and Region

Having explored the relationship between health expenditure and adult education, we then explored the relationship between health expenditure and NEET rates of different countries (Fig. 9). We expect compulsory health expenditure to be negatively associated with NEET rates.

In Fig. 9, each box represents a country or region; the size of the box indicates the extent of compulsory health expenditure such that a larger box implies that the country has greater compulsory health expenditure. The intensity of the color of the box represents the NEET rate such that the darker color implies a higher NEET rate. Turkey has the highest NEET rate with low health expenditure. Most European countries such as France, Belgium, Sweden, and Norway have low NEET rates and high health expenditure. The chart shows a general association between low compulsory health expenditure and high NEET rates. The relationship, however, is not consistent, as there are countries with high NEET and high health expenditures. Our suggestion is for most countries to improve the social education for the youth through free training programs and other means to effectively improve the public health while they attempt to raise the compulsory expenditure.

### Distribution of life expectancy at birth and tertiary enrollment rate

**Fig. 10:**
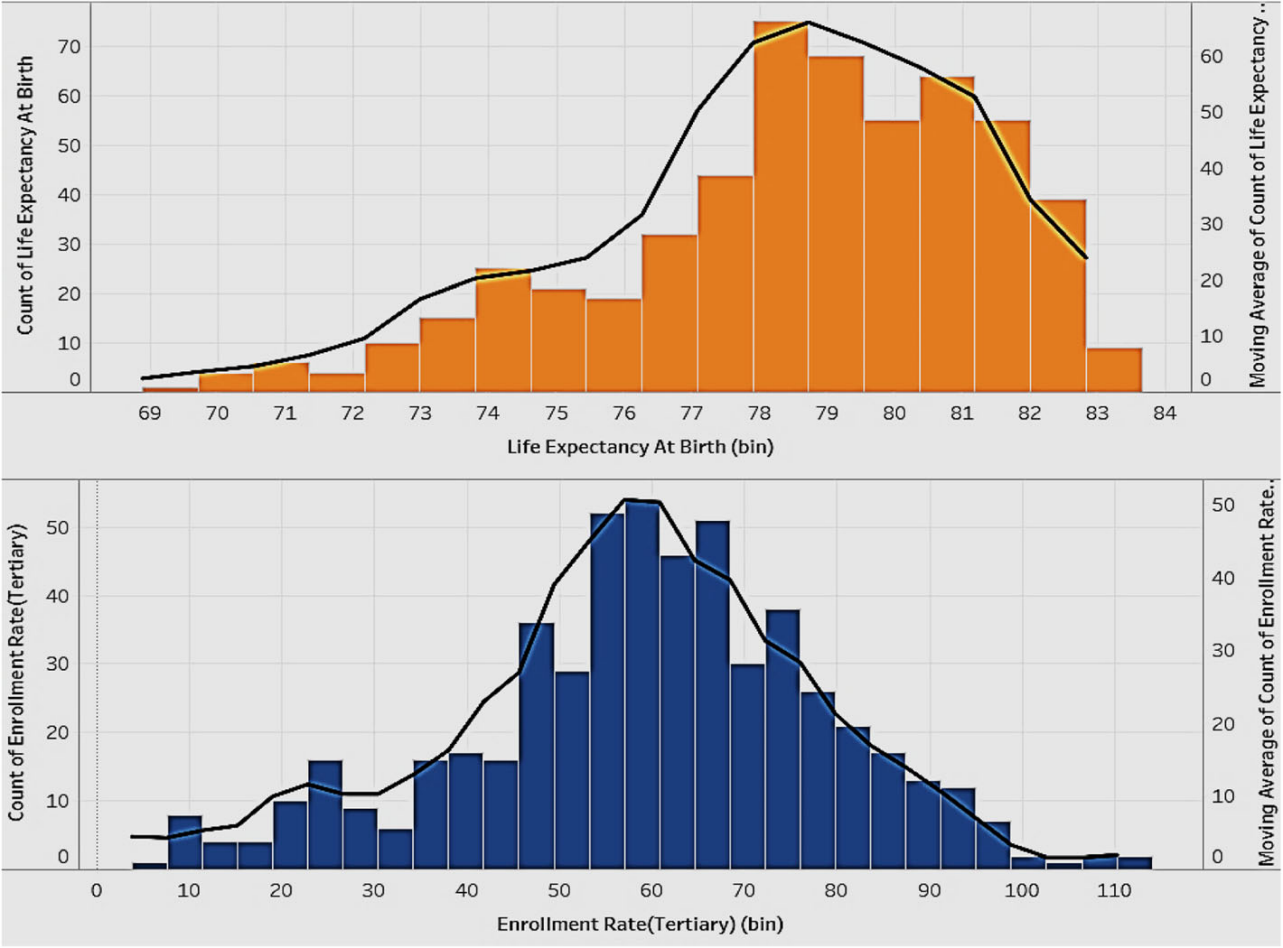
Distribution of Life Expectancy at Birth (years) and Tertiary Enrollment Rate

The distribution of enrollment rate (tertiary) and life expectancy of all the countries in the sample can give an idea of the current status of both education and health (Fig. 10). We expect these to be positively associated.

Figure 10 shows two histograms with the lines representing the distribution of life expectancy at birth and the tertiary enrollment rate of all the countries. The distribution of life expectancy at birth is skewed right, which means most of the countries have quite a high life expectancy and there are few countries with a very low life expectancy. The tertiary enrollment rate has a good distribution, which is closer to a normal distribution. Governments of countries with an extremely low life expectancy should try to identify the cause of this problem and take actions in time to improve the overall national health.

### Comparison of adult education levels and deaths from Cancer at various levels of GDP per capita

**Fig. 11:**
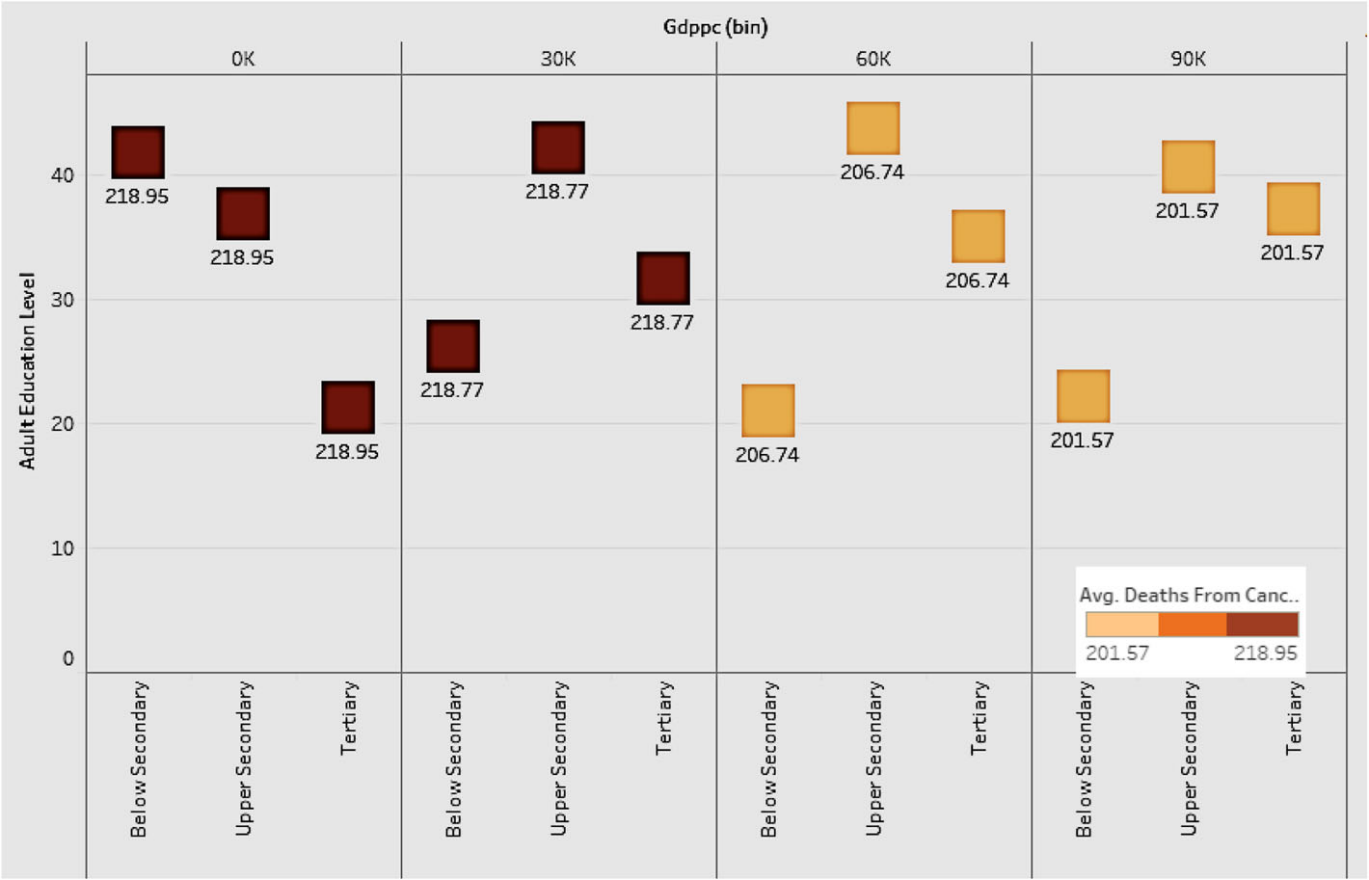
Comparison of Adult Education Levels and Deaths from Cancer at various levels of GDP per capita

We wanted to see if various levels of GDP per capita influence the levels of adult education and deaths from cancer in countries (Fig. 11).

Figure 11 shows the distribution of various adult education levels for countries by groups of GDP per capita. The plot shows that as GDP grows, the level of below-secondary adult education becomes lower, and the level of tertiary education gets higher. The upper-secondary education level is constant among all the groups. The implication is that tertiary education is the most important factor among all the education levels for a country to improve its economic power and health level. Countries should therefore focus on tertiary education as a driver of economic development. As for deaths from cancer, countries with lower GDP have higher death rates, indicating the negative association between economic development and deaths from cancer.

### Distribution of infant mortality rates by continent

**Fig. 12:**
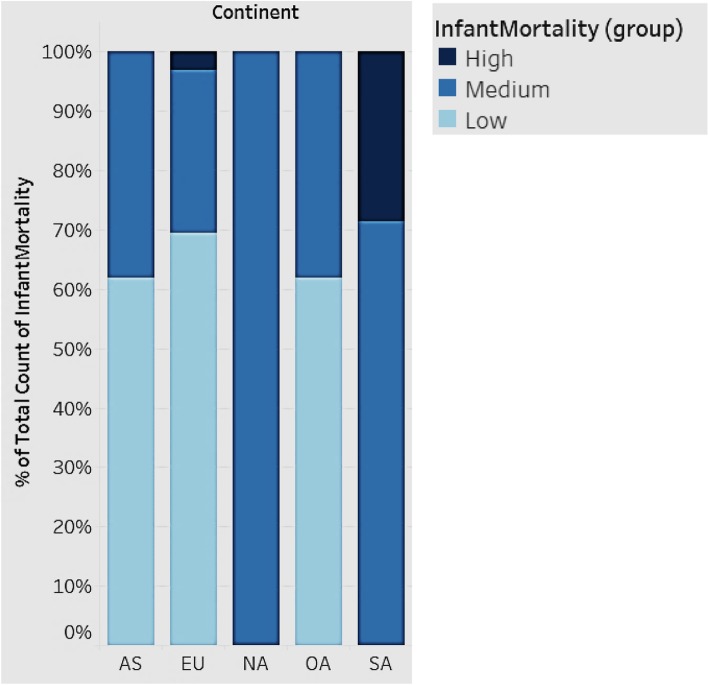
Distribution of Infant Mortality rates by Continent

Infant mortality is an important indicator of a country’s health status. Figure 12 shows the distribution of infant mortality for the continents of Asia, Europe, Oceania, North and South America. We grouped the countries in each continent into high, medium, and low, based on infant mortality rates.

In Fig. 12, each bar represents a continent. All countries fall into three groups (high, medium, and low) based on infant mortality rates. South America has the highest infant mortality, followed by Asia, Europe, and Oceania. North America falls in the medium range of infant mortality. South American countries, in general, should strive to improve infant mortality. While Europe, in general, has the lowest infant mortality rates, there are some countries that have high rates as depicted.

### Association between child vaccination rates and NEET rates

**Fig. 13:**
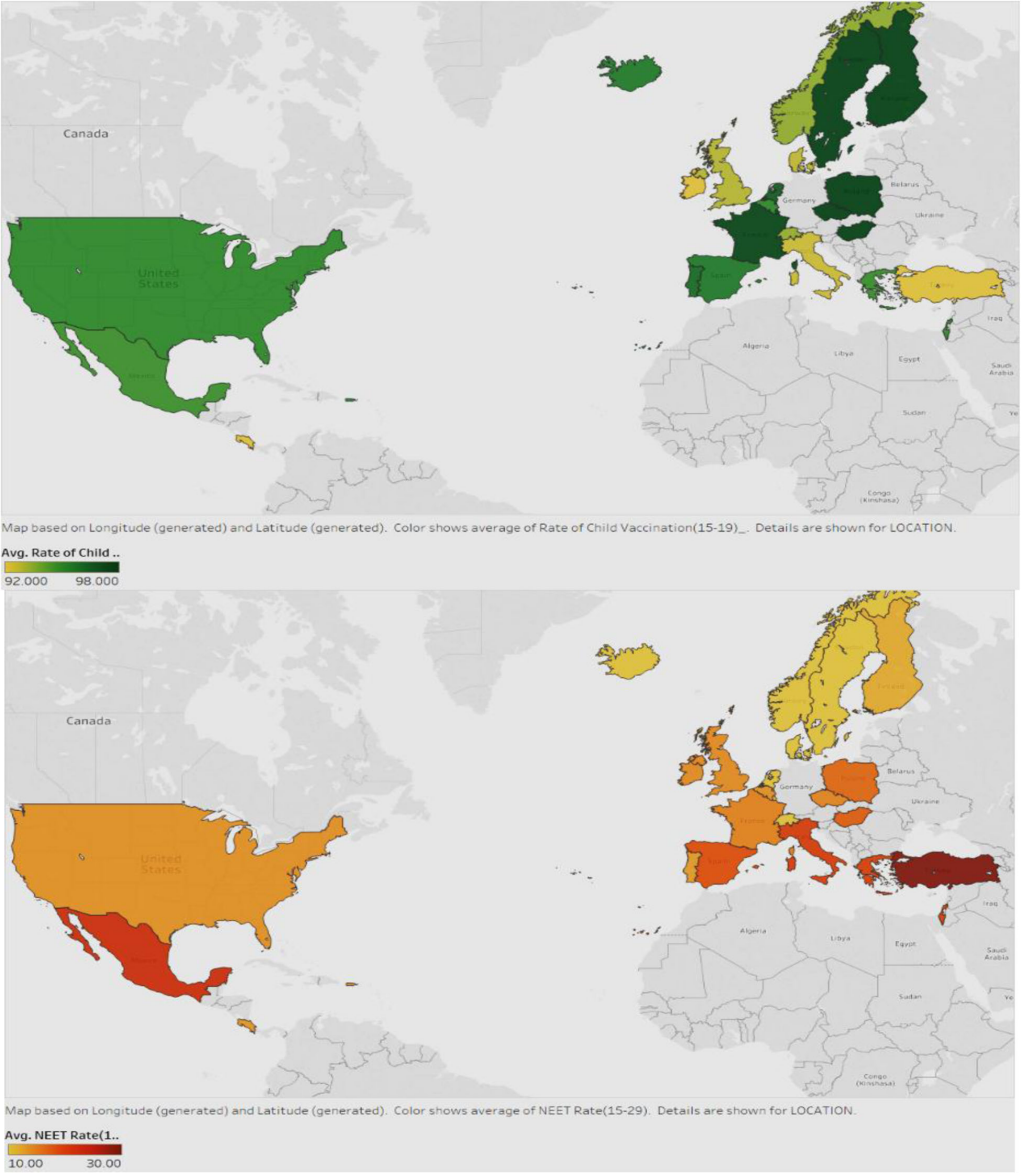
Association between Child Vaccination Rates and NEET rates

We looked at the association between child vaccination rates and NEET rates in various countries (Fig. 13). We expect countries that have high NEET rates to have low child vaccination rates.

Figure 13 displays the child vaccination rates in the first map and the NEET rates in the second map, for all countries. The darker green color shows countries with higher rates of vaccination and the darker red represents those with higher NEET rates. It can be seen that in general, the countries with lower NEET also have better vaccination rates. Examples are USA, UK, Iceland, France, and North European countries. Countries should therefore strive to reduce NEET rates by enrolling a good proportion of the youth into initiatives or programs that will help them be more productive in the future, and be able to afford preventive healthcare for the families, particularly, the children.

### Average smoking rate in different continents over time

**Fig. 14:**
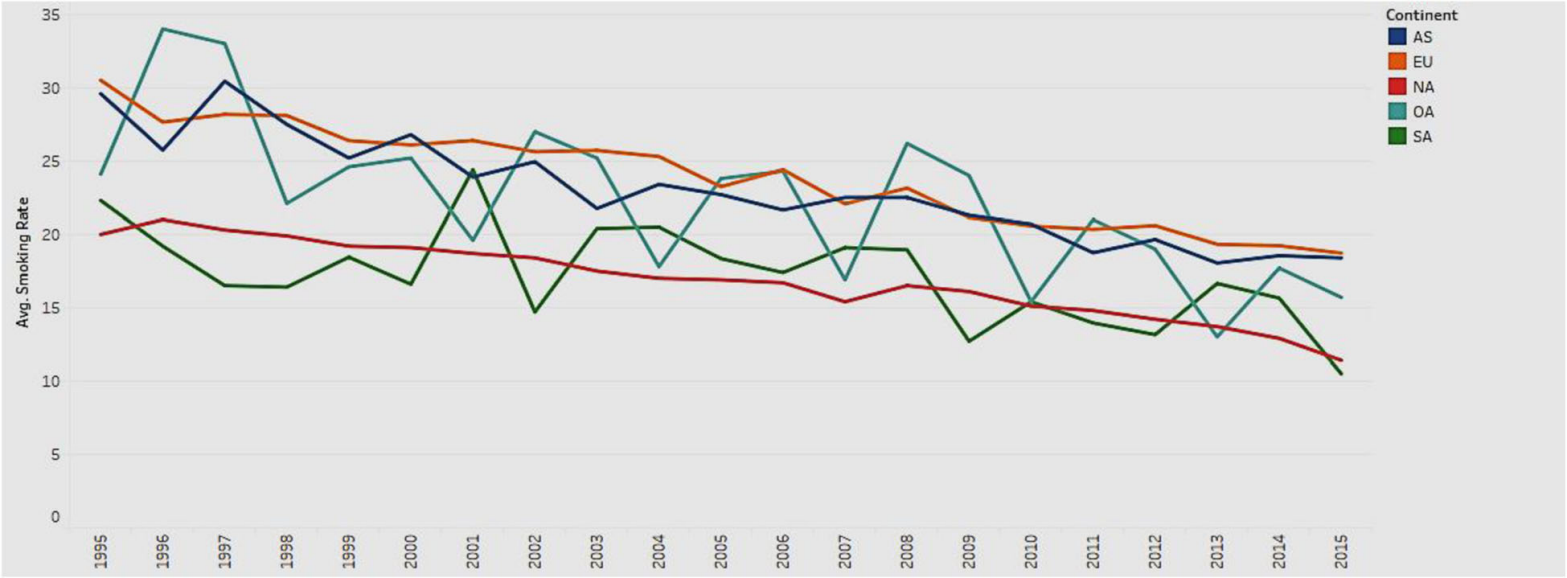
Trend of average smoking rate in different continents from 1995 to 2015

We compared the trend of average smoking rate for the years 1995–201 for the continents in the sample (Fig. 14).

Figure 14 depicts the line charts of average smoking rates for the continents of Asia, Europe, Oceania, North and South America. All the lines show an overall downward trend, which indicates that the average smoking rate decreases with time. The trend illustrates that people have become more health conscious and realize the harmful effects of smoking over time. However, the smoking rate in Europe (EU) is consistently higher than that in other continents, while the smoking rate in North America (NA) is consistently lower over the years. Governments in Europe should pay attention to the usage of tobacco and increase health consciousness among the public.

### Association between adult education levels and deaths from Cancer

**Fig. 15:**
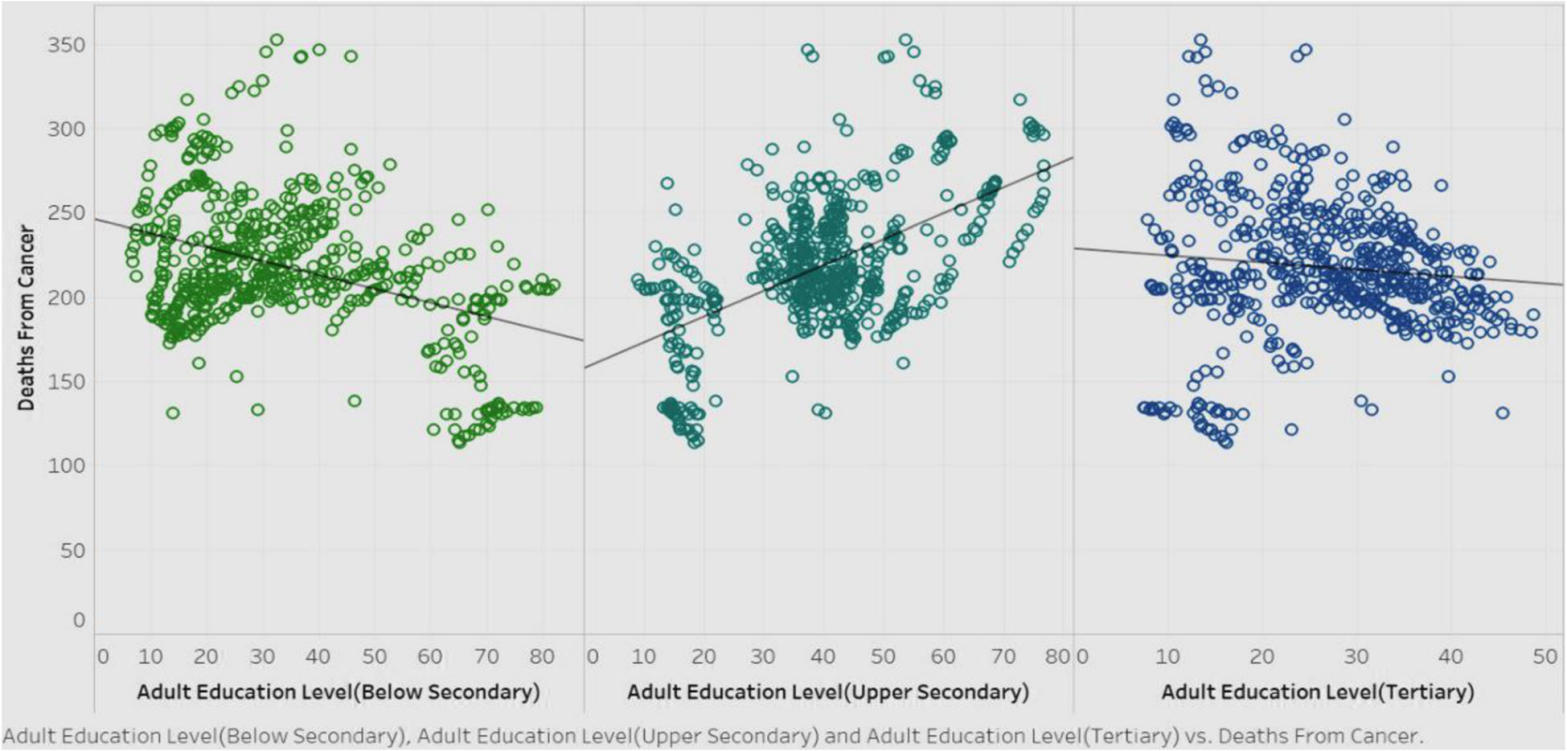
Association of deaths from cancer with adult education levels

We explored if adult education levels (below-secondary, upper-secondary, and tertiary) are associated with deaths from cancer (Fig. 15) such that higher levels of education will mitigate the rates of deaths from cancer, due to increased awareness and proactive health behavior.

Figure 15 shows the correlations of deaths from cancer among the three adult education levels, for all countries in the sample. It is obvious that below-secondary and tertiary adult education levels have a negative correlation with deaths from cancer, while the upper-secondary adult education level shows a positive correlation. Barring upper-secondary results, we can surmise that in general, as education level goes higher, the deaths from cancer will decrease. The rationale for this could be that education fosters more health awareness and encourages people to adopt healthy behavioral practices. Governments should therefore pay attention to frame policies that promote education. However, the counterintuitive result of the positive correlation between upper-secondary levels of adult education with the deaths from cancer warrants more investigation.

**Fig. 16:**
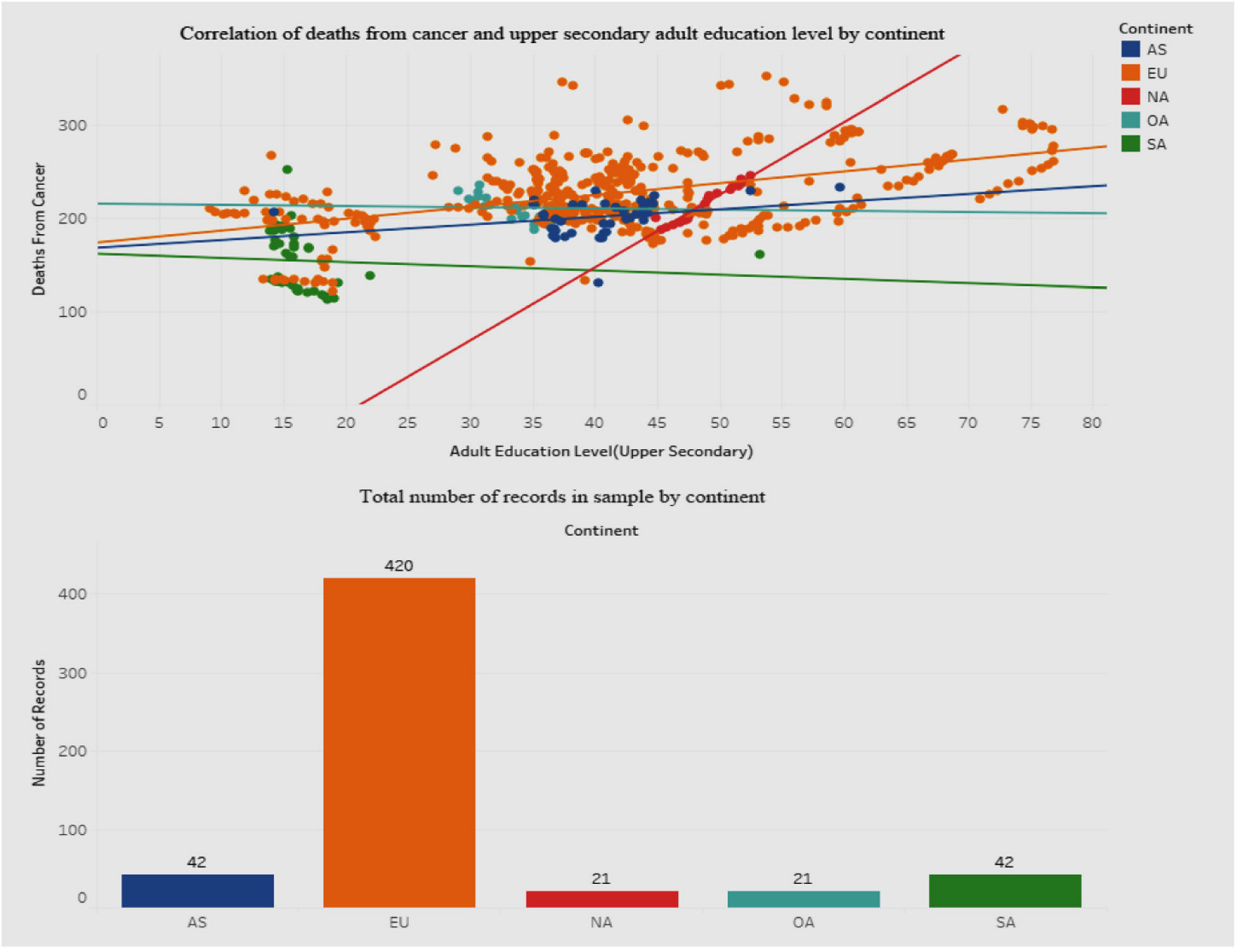
Association between deaths from cancer and adult education level-upper secondary

We drilled down further into the correlation between the upper-secondary education level and deaths from cancer. Figure 16 shows this correlation, along with a breakdown of the total number of records for each continent, to see if there is an explanation for the unique result.

Figure 16 shows a dashboard containing two graphs - a scatterplot of the correlation between deaths from cancer and education level, and a bar graph showing the breakdown of the total sample by continent. We included a breakdown by continent in order to explore variances that may clarify or explain the positive association for deaths from cancer with the upper-secondary education level. The scatterplot shows that for the European Union (EU) the points are much more scattered than for the other continents. Also, the correlation between deaths and education level for the EU is positive. The bottom bar graph depicts how the sample contains a disproportionately high number of records for the EU than for other continents. It is possible that this may have influenced the results of the correlation. The governments in the EU should investigate the reasons behind this phenomenon. Also, we defer to future research to explore this in greater detail by incorporating other socioeconomic parameters that may have to be factored into the relationship.

### Association between average tertiary school life expectancy and health expenditure

**Fig. 17:**
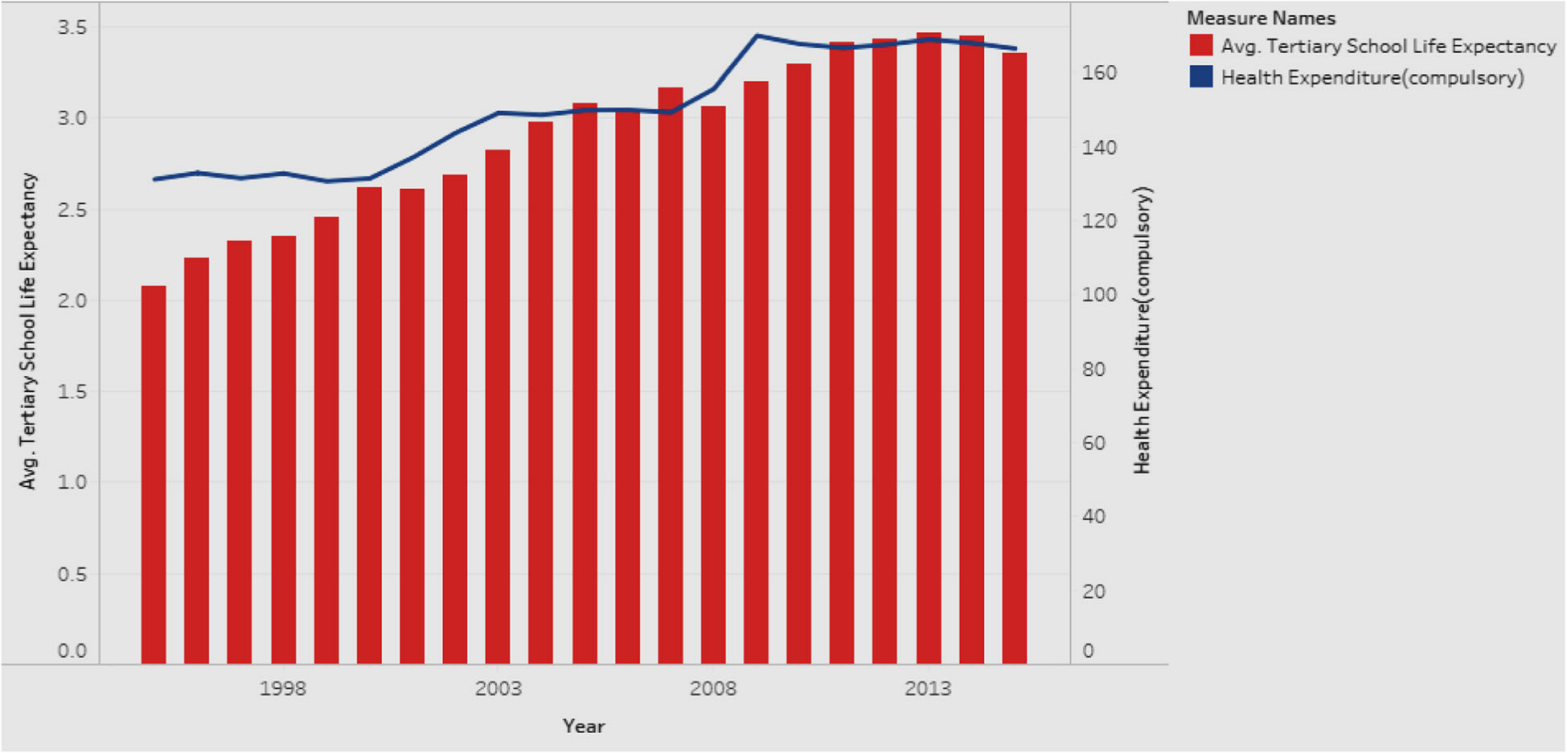
Association between Average Tertiary School Life Expectancy and Health Expenditure

We moved our focus to the trends of tertiary school life expectancy and health expenditure from 1995 to 2015 (Fig. 17) to check for positive associations.

Figure 17 is a combination chart explaining the trends of tertiary school life expectancy and health expenditure, for all countries in the sample. The rationale is that if there is a positive association between the two, it would be worthwhile for the government to allocate more resources towards health expenditure. Both tertiary school life expectancy and health expenditure show an increase over the years from 1995 to 2015. Our additional analysis shows that they continue to increase even after 2015. Hence, governments are encouraged to increase the health expenditure in order to see gains in tertiary school life expectancy, which will have positive implications for national health. Given that the measured effects of education are large, investments in education might prove to be a cost-effective means of achieving better health.

## Discussion

Our results reveal how interlinked education and health can be. We show how a country can improve its health scenario by focusing on appropriate indicators of education. Countries with higher education levels are more likely to have better national health conditions. Among the adult education levels, tertiary education is the most critical indicator influencing healthcare in terms of infant mortality, life expectancy, child vaccination rates, and enrollment rates. Our results emphasize the role that education plays in the potential years of life lost, which is a measure that represents the average years a person would have lived had he/she not died prematurely. In addition to mortality rate, an economy needs to consider this indicator as a measure of health quality.

Other educational indicators that are major drivers of health include school life expectancy, particularly at the tertiary level. In order to improve the school life expectancy of the population, governments should control the number of youths ending up unemployed, dropping out of school, and without skills or training (the NEET rate). Education allows people to gain skills/abilities and knowledge on general health, enhancing their awareness of healthy behaviors and preventive care. By targeting promotions and campaigns that emphasize the importance of skills and employment, governments can reduce the NEET rates. And, by reducing the NEET rates, governments have the potential to address a broad array of vulnerabilities among youth, ranging from unemployment, early school dropouts, and labor market discouragement, which are all social issues that warrant attention in a growing economy.

We also bring to light the health disparities across countries and suggest implications for governments to target educational interventions that can reduce inequalities and improve health, at a macro level. The health effects of education are at the grass roots level - creating better overall self-awareness on personal health and making healthcare more accessible.

## Scope and limitations

Our research suffers from a few limitations. For one, the number of countries is limited, and being that the data are primarily drawn from OECD, they pertain to the continent of Europe. We also considered a limited set of variables. A more extensive study can encompass a larger range of variables drawn from heterogeneous sources. With the objective of acquiring a macro perspective on the education–health association, we incorporated some dependent variables that may not traditionally be viewed as pure health parameters. For example, the variable potential years of life lost is affected by premature deaths that may be caused by non-health related factors too. Also there may be some intervening variables in the education–health relationship that need to be considered. Lastly, while our study explores associations and relationships between variables, it does not investigate causality.

## Conclusions and future research

Both education and health are at the center of individual and population health and well-being. Conceptualizations of both phenomena should go beyond the individual focus to incorporate and consider the social context and structure within which the education–health relationship is embedded. Such an approach calls for a combination of interdisciplinary research, novel conceptual models, and rich data sources. As health differences are widening across the world, there is need for new directions in research and policy on health returns on education and vice versa. In developing interventions and policies, governments would do well to keep in mind the dual role played by education—as a driver of opportunity as well as a reproducer of inequality [36]. Reducing these macro-level inequalities requires interventions directed at a macro level. Researchers and policy makers have mutual responsibilities in this endeavor, with researchers investigating and communicating the insights and recommendations to policy makers, and policy makers conveying the challenges and needs of health and educational practices to researchers. Researchers can leverage national differences in the political system to study the impact of various welfare systems on the education–health association. In terms of investment in education, we make a call for governments to focus on education in the early stages of life course so as to prevent the reproduction of social inequalities and change upcoming educational trajectories; we also urge governments to make efforts to mitigate the rising dropout rate in postsecondary enrollment that often leads to detrimental health (e.g., due to stress or rising student debt). There is a need to look into the circumstances that can modify the postsecondary experience of youth so as to improve their health.

Our study offers several prospects for future research. Future research can incorporate geographic and environmental variables—such as the quality of air level or latitude—for additional analysis. Also, we can incorporate data from other sources to include more countries and more variables, especially non-European ones, so as to increase the breadth of analysis. In terms of methodology, future studies can deploy meta-regression analysis to compare the relationships between health and some macro-level socioeconomic indicators [13]. Future research should also expand beyond the individual to the social context in which education and health are situated. Such an approach will help generate findings that will inform effective educational and health policies and interventions to reduce disparities.

## Data Availability

The dataset analyzed during the current study is available from the corresponding author on reasonable request.

## Abbreviations

FCT: Fundamental Cause Theory
HCT: Human Capital Theory
NEET: Not in Employment, Education, or Training
OECD: Organization for Economic Cooperation and Development
SES: Socio-economic status
